# Using Community Advisory Boards to Reduce Environmental Barriers to Health in American Indian Communities, Wisconsin, 2007–2012

**DOI:** 10.5888/pcd11.140014

**Published:** 2014-09-18

**Authors:** Alexandra K. Adams, Jamie R. Scott, Ron Prince, Amy Williamson

**Affiliations:** Author Affiliations: Jamie R. Scott, Ron Prince, Amy Williamson, University of Wisconsin School of Medicine and Public Health, Madison, Wisconsin.

## Abstract

**Background:**

American Indian communities have a high prevalence of chronic diseases including diabetes, obesity, cardiovascular disease, and cancer. Innovative community-based approaches are needed to identify, prioritize, and create sustainable interventions to reduce environmental barriers to healthy lifestyles and ultimately improve health.

**Community Context:**

Healthy Children, Strong Families was a family-based and community-based intervention to increase healthy lifestyles on Wisconsin American Indian reservations. This intervention arose from a long-standing partnership between University of Wisconsin researchers and 3 of these American Indian communities.

**Methods:**

In each community, community advisory boards (CABs) were established by the residents and university partners. CAB meetings were open and held at various times and locations to increase member participation. CABs featured continual, snowball recruitment; internal and external expert consultation; and coordination with standing tribal committees. Meetings initially focused on understanding community supports for and barriers to healthy lifestyles but quickly turned toward community action for change.

**Outcome:**

CAB interventions decreased environmental barriers to health at each site and improved options for healthy lifestyle choices. Over 5 years, 71 CAB meetings occurred with a total of 1,070 participants. Successful CAB interventions included planting community gardens and an apple orchard, conducting gardening and canning workshops, instituting food-related policies and dog control regulations, building an environmentally friendly playground, and providing access to recreational facilities. The CABs are now self-sustaining.

**Interpretation:**

CABs can be highly effective action teams capable of improving community environments. Our experience shows that academic researchers can partner with community residents to generate programs and policies that will expand access to local food, increase people’s choices for engaging in physical activity, and encourage local policy changes that improve overall community health.

## Background

American Indian (AI) people have the highest rates of diabetes (1) and cardiovascular disease (1,2) and the poorest progress in reducing cancer rates of any ethnic or racial group in the United States (3). Indian Health Service data reveal that among all AIs, those in the Bemidji area (Minnesota, Wisconsin, and Michigan) have the highest rates of cardiovascular disease and the second highest rates of diabetes nationally (4). Interventions designed in collaboration with communities are needed to increase healthy lifestyle behaviors, prevent chronic disease, and improve health (5). Few studies have focused on environmental barriers to health in rural reservation communities, although most tribes are working to improve conditions locally.

Since 2001, we have partnered with northern Wisconsin tribal communities using community-based participatory research (CBPR) to design and implement effective interventions to reduce childhood obesity. “Healthy Children, Strong Families” (HCSF) was a 5-year randomized intervention funded by the National Institutes of Health (2006–2011) and the Wisconsin Partnership Program (2004–2008) as a healthy lifestyles program for children aged 2 to 5 years and their families (6,7). We describe HCSF’s Supportive Communities component, which worked with 3 tribal communities to develop community advisory boards (CABs) to assess and eliminate environmental barriers to health.

## Community Context

CABs were developed with the Menominee, Lac du Flambeau, and Bad River communities in rural northern Wisconsin that have AI tribes ranging from 2,000 to 7,000 members. Our previous research found that 47% of AIs aged 2 to 5 years living in these communities were overweight or obese and 79% of parents of these children were also overweight or obese (6). We found significant barriers to healthy lifestyles such as lack of access to fresh produce, poor play spaces, and lack of safe exercise areas (8). Such environments may also be “food deserts” where there is low access to fresh fruits and vegetables and other healthy foods, with significant driving distances to healthy food outlets (9).

## Methods

### Community engagement

To support the individual and family change promoted by the HCSF childhood obesity intervention (6,8), university and tribal partners organized a CAB with each of the 3 tribes. The goal of each CAB was to reduce environmental barriers to healthy diet and exercise. By engaging tribal communities in forming their own CAB, our aim was to identify, prioritize, and create sustainable community interventions to reduce these barriers and ultimately improve community health. A university-based facilitator (non-AI) was hired to help launch and coordinate the CABs. CAB approval was obtained from each tribal council. We invited each CAB to review this manuscript and incorporated their comments.

### Building CAB membership

Four outreach strategies were used to ensure community-wide representation, build membership, and increase community engagement in CAB activities. First, the facilitator approached standing tribal committees and invited members to participate in the CAB. The facilitator also asked these individuals to recommend and invite additional community members. Second, one-on-one networking and snowball techniques were used. The facilitator met with many individuals, resulting in an e-mail and address list of 70 to 95 contacts for each CAB. Third, the facilitator created “save the date” meeting notices with an open meeting invitation; these notices were disseminated by mail, on community bulletin boards, and in tribal newspapers. This approach brought various community members together, including those who traditionally did not participate in tribal meetings. Fourth, continued open invitations and member recruitment were used to enhance membership throughout CAB operation.

The composition of each CAB varied slightly and reflected the individual tribal community. We did not limit membership, although other research has shown that new partnerships should start small and involve a few highly regarded community-based organizations (10). We initially compiled a list of more than 100 occupations to be represented on the CABs but soon chose not to limit membership by livelihood. The university partners did not know nor want to presume the specific issues or outcomes that would be important to each CAB. From the onset, CAB membership included community, academic, and tribal partner stakeholders (11).

### CAB operations

The facilitator (the same person for all 3 CABs) organized meetings, traveled to each site for monthly meetings, maintained e-mail lists, and spent considerable time connecting one-on-one with potential and current CAB members. Because the sites were 3 to 5.5 hours by car from the university, the facilitator scheduled meetings sequentially to avoid meeting conflicts and reduce total travel time. Over time, all of the partners found that facilitation by a non-AI, someone who had no tribal political affiliation, was important in encouraging open communication and political neutrality. That the facilitator was an academic partner also lent legitimacy and credibility to meetings.

Each CAB decided to make the meetings open, not pay people for participating, and not reimburse travel expenses. CAB meetings occurred at varied times of day to accommodate schedules and always included a healthy meal (sometimes incorporating traditional foods). The facilitator prepared an agenda for each meeting, but quorum was not required to proceed with business. At the start of each meeting, the facilitator gave a brief overview of the CAB’s history and mission to orient new members.

In advance of each meeting, CAB members or the facilitator met with new members to provide background, including information on activities and successes, a written synopsis of what had been accomplished so far, and the upcoming meeting’s agenda. Project funds paid for meeting costs and the facilitator’s travel costs.

A key component of CAB operations was planning for transition to local coordination before the end of the 5-year grant. At each site during the third and fourth years, the academic facilitator selected a community facilitator with the CAB’s help. The two then co-organized meetings, and at some meetings the academic facilitator was present by telephone only, to recognize the importance of the local leader.

### Phases of CAB work

A key activity of early meetings was to brainstorm to identify local environmental supports that made it easy for people to make healthy diet and physical activity choices and to identify environmental barriers that impeded healthy choices. Initial lists of supports and barriers were unique to each community but had numerous common themes. The Box details environmental supports noted by all CABs, including strong cultural identity, natural resources, family ties, and tribal agency services. Barriers to healthy nutrition included limited access to fresh fruits and vegetables, easily available processed foods, and alcohol and sugared drinks at local convenience stores. Barriers to physical activity included lack of sidewalks, loose dogs, and unsafe areas. Similar barriers were combined to simplify focus and then prioritized by perceived importance. Through this process, issues that could be influenced by interventions, both short-term and long-term, were moved to the top of the list and addressed.

**Box. Environmental Supports for and Barriers to Healthy Lifestyles Cited by 3 Wisconsin Tribal Community Advisory Boards**

Supports

**Location and natural resources:** Hunting, fishing, boating, hiking, harvesting opportunities

**Strong cultural awareness and participation in traditional activities:** Powwows, sweat lodges, hunting, water walks, naming ceremonies, spirituality, sugar camp, rice harvests, traditional foods, funerals, community feasts, festivals

**Family support:** Immediate and extended, across generations, co-raising grandchildren, nieces and nephews, individual assets

**Tribal programs and agencies:** Housing, food distribution, Boys and Girls Clubs, health committee, early childhood, language and culture, legislature, elder housing and food service, mental health services, transit, recreation, police

**Tribal clinics and programs:** Special Supplemental Nutrition Program for Women, Infants, and Children; diabetes education; wellness and dental programs: doulas; school health education; prevention programs; walking groups

**Schools:** Tribal and public sports programs, Head Start, day care

**Collaborative partners:** State, university, academic experts, and researchers; University of Wisconsin County Extension; state and county police; AmeriCorps workers

**Striving for healthier futures:** Tribal strategic plans, agency objectives, health policies in place

Barriers

**Historic trauma**: Mistrust of European culture and non-AI researchers because of US government’s actions during the assimilation and allotment era; nonsovereign nation judicial system

**Poverty and limited local economic opportunities:** Lack of jobs, lack of affordable housing

**Stress: **Issues related to poverty; single-parent households; inequity; racism; life expectancy; teen pregnancy rate; substance abuse; physical, emotional, and mental illness; gambling; extended family living within one home; daily survival

**Time constraints:** Balancing multiple jobs, shift work, traveling round-trip off reservation for work, caring for family

**Safety:** Issues related to poverty, crime, automobile accidents, domestic abuse, high recidivism rates, suicide, lack of safe places to walk, play, exercise, loose dogs, wild animals

**Lack of intra-reservation communication:** No daily tribal newspapers, no radio stations, lack of computer access

**Lack of knowledge about healthy eating and traditional foods:** Limited knowledge of gardening or of gathering, preparing, and preserving food.

**Access to healthy foods:** Self-identified as living in food deserts, lack of grocery stores, and only convenience stores on reservations.

**Figure 1:**
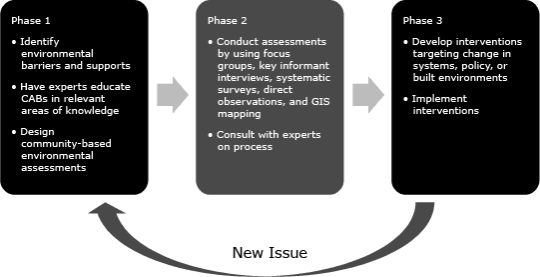
Community advisory board (CAB) work flow for creation of community-based interventions to promote healthy lifestyles, Wisconsin, 2007–2012. Abbreviation: GIS, geographic information system.

Initially, we envisioned CAB work to be done in 3 phases (Figure 1). In Phase 1, CABs reviewed supports and barriers, prioritized barriers, and discussed potential intervention options. In Phase 2, CABs conducted community assessments of barriers, collecting data and expert opinions as needed, which led to action to address the barriers in Phase 3. The timeframe of this assessment and action cycle varied but, in all locations, moved to the intervention phase more quickly than anticipated. Phase 2 assessments were largely either already in place, not done, or done rapidly so the CAB could move quickly into action.

Invited experts helped the CABs understand the nature of environmental barriers in each community while clarifying options for intervention. Experts included academic specialists in nutrition and physical activity and community experts in gardening, nutrition, and law enforcement. CAB members gathered information on specific topics (such as current tribal policies, jurisdiction, and future guest experts) and reported back to the CAB. The CAB then designed interventions and took action steps to remove or reduce a barrier (sometimes through a designated subcommittee). Each year, this cycle was repeated many times with different barriers. To facilitate policy change, each CAB included members who were on various tribal committees (see Outcomes) and who shared information about CAB initiatives with other committee members. The facilitator and/or CAB members also reported on CAB activities annually to each tribal council.

CABs sustained momentum by regularly reviewing successes and selecting the next issues to address. Subcommittees took ownership of specific barriers. The facilitator tracked progress and shared ideas and successes with the other CABs. At each meeting, CABs decided whether the CAB was ready to move on to another barrier. With new participants joining longstanding members, each CAB’s focus continually shifted, as membership assessed, advised, and reprioritized initiatives. CAB members were surveyed annually to evaluate CAB function, engagement, and collaboration. Because of the high level of engagement measured at baseline, survey results did not change significantly over time.

## Outcomes

The overarching goal of the CABs was to reduce environmental barriers to healthy diet and exercise in the 3 tribal communities. We measured impact in terms of process (meeting metrics) and outcomes (interventions, inter-CAB sharing, academic engagement). We collected data from document review and observation.

### CAB meeting metrics

Although a core group of members attended each CAB meeting from the onset, meeting attendance was fluid, totaling more than 1,070 regular and new members over 5 years for the 3 CABs and an average meeting attendance of 19 (Table 1). This was much greater than the partners’ prelaunch assumption that CABs would include 5 to 10 standing members.

**Table 1:** Community Advisory Board (CAB) Process Measures for Each Tribal Site, Wisconsin, 2007–2012

Tribal CAB	Total No. of CAB Meetings	Total No. of CAB Attendees^a^	Average Meeting Attendance	No. of Subcommittees Working on Specific Initiatives
Bad River	24	354	18	3
Lac du Flambeau	19	181	19	1
Menominee	28	535	19	1
Total	71	1,070	19	5

a Represents composite of continuing and new members.

Meetings were regularly attended by community members, academic experts, and individuals representing tribal planning, education, transportation, health clinics, nutrition and disease prevention programs, human resources, environmental services, commerce, housing, food distribution, and recreation. Other members represented tribal and county extension, Boys and Girls Clubs, local media, chambers of commerce, casino employees, and local colleges. Members represented various occupational, professional, political, familial, cultural, and personal perspectives and often represented multiple roles and types of expertise. CAB members who had not previously participated in tribal meetings reported that they appreciated being part of the process.

### CAB interventions

**Figure 2:**
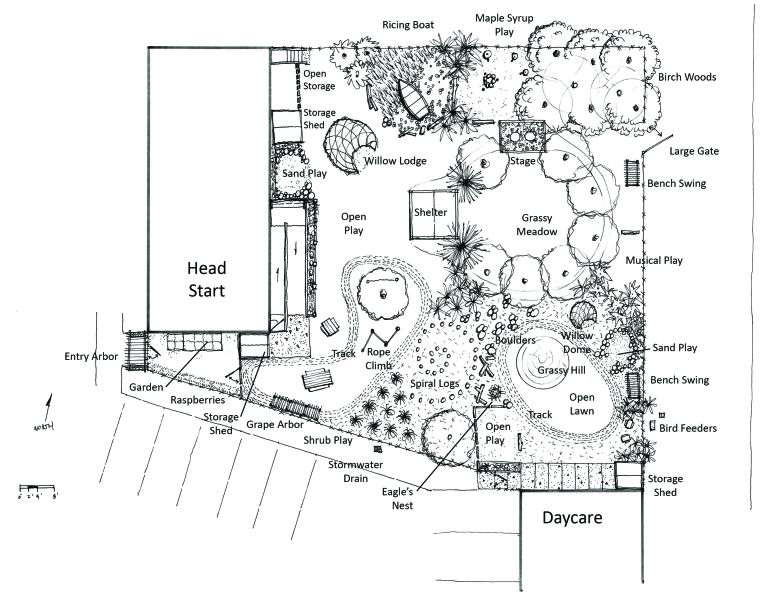
Early plan for Bad River Playground, Wisconsin. University of Wisconsin Associate Professor Sam Dennis (landscape architecture) worked with tribal elders to design the playground at the Bad River Head Start following principles of natural playgrounds, which encourage imaginative and creative play.

**Figure 3:**
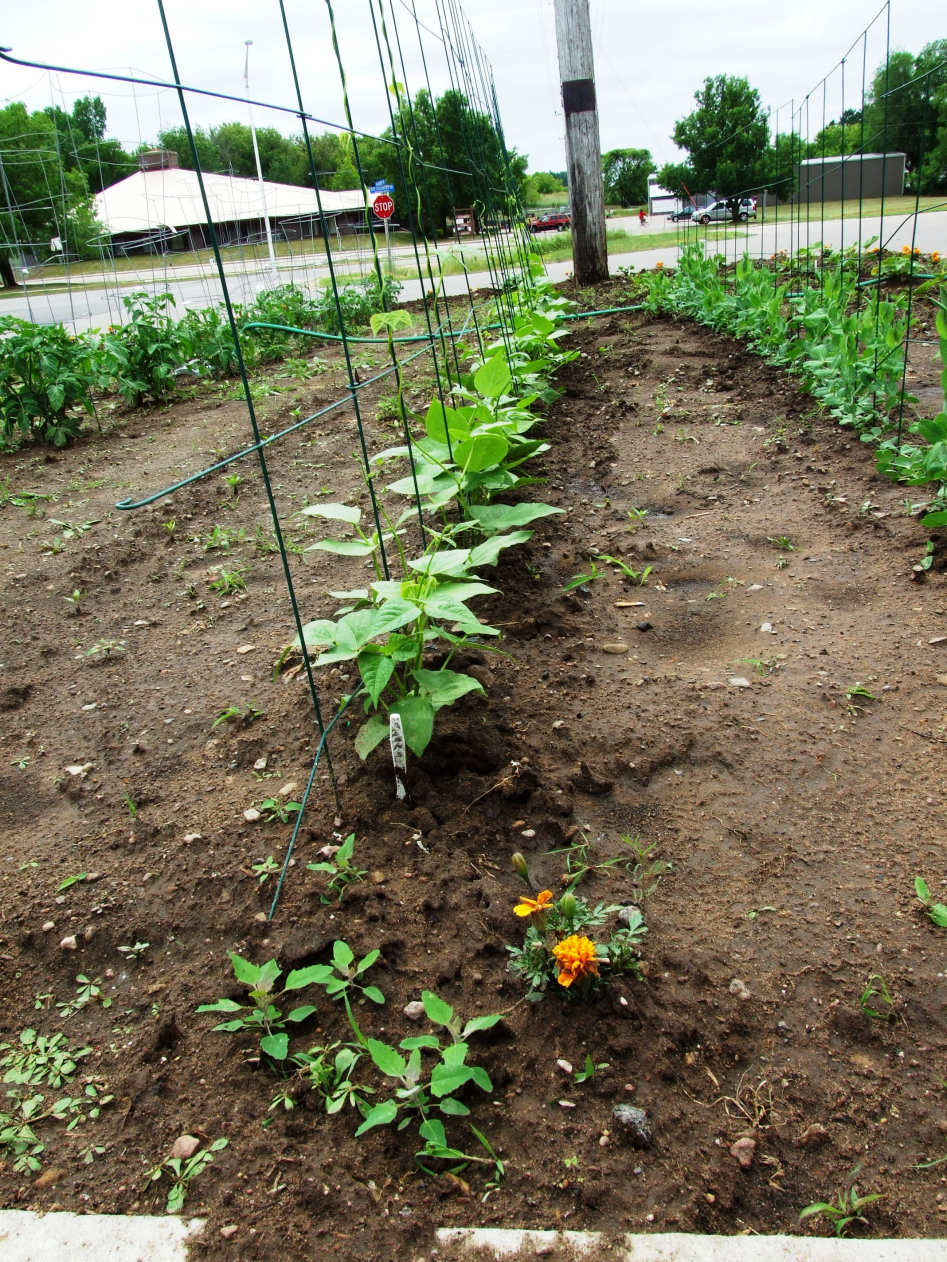
A garden planted on the grounds of the Menominee Tribal Food Distribution agency, Wisconsin. Both public and private gardens were planted.

Each CAB completed varying numbers and types of interventions (Table 2). Timeline for intervention completion varied and often depended on who was in attendance when a barrier was identified; who made the barrier a personal project, brought new members to the CAB, or volunteered to lead the issue subcommittee; or who had the political power to make it an agency or tribal priority. One intervention was introduced and completed in 4 hours (police and animal rescue panel to address safety issues of wandering dogs). Another lasted 18 months from planning to completion (playground project [Figure 2] requiring academic team travel and assistance, numerous tribal agency and community volunteer person-hours, installation during inclement weather, and secondary reconfiguration). A third intervention is entering its sixth year of operation (community and individual gardening); this highly active subcommittee has a structured short and long-term vision for gardening in the community (Figure 3).

**Table 2:** Environmental Barriers and Community Solutions for Healthy Lifestyles in American Indian Communities, Wisconsin, 2007–2012^a^

Tribe/Identified Barrier	Intervention	Solutions/Impact
**Menominee**		
Loose dogs inhibit people from walking for exercise	Police and animal rescue panel discussed protocol and answered questions	Addition of another dog catcher More dogs neutered, spayed, and micro-chipped Dogs rescued by breed-specific adoption agencies around state
No grocery store on reservation; limited access to fresh produce	Gardening subcommittee formed Raised beds constructed Community and individual gardens tilled and planted Plants and seeds distributed Gardening workshops offered Transportation to farmers market provided	Gardening Subcommittee entering sixth year of existence 8 community gardens planted 63 individual gardens planted 27 gardening and food preservation workshops conducted and attended by 598 participants Subcommittee attended national gardening workshop Apple orchard installed at school district site Grocery store built on reservation Teaching kitchen installed
Lack of sidewalks and involvement in physical activity	CAB, UW, and tribal partners sponsored physical activity and gardening stations	First lady Michele Obama’s Inaugural National Let’s Move in Indian Country event held
**Lac du Flambeau**		
Lack of physical activity and healthy nutritional awareness	CAB and community members organized and conducted physical activity and health stations	Let’s Move event at K-8 school
Limited knowledge of gardening	Designed game and purchased gardening supply prizes	Elders Gardening (Bingo) event (attended by 45 men and women)
Limited access to fresh produce and few gardens because of limited knowledge and short growing season	Menominee CAB gardening expert and subcommittee member presented steps and timeline from Menominee gardening initiative	Intergenerational container gardening workshops held, and tomato plants distributed
**Bad River**		
Lack of safe play spaces	Elders focus group reminisced about outdoor play Designed playground incorporating elders’ suggestions	Install award-winning environmentally friendly and cultural playground; for example, willow lodge, ricing canoe (used for harvesting wild rice), and climbing logs
Unsafe Lake Superior beach access	UW landscape architect student and tribal Department of Natural Resources solicited CAB suggestions and designed safe tiered landscaping	Installation of landscaping and indigenous plants for beach accessibility by people of all abilities
Limited knowledge of gardening; short growing season	CAB members attended gardening workshops	CAB Sustainable Gardening Subcommittee formed
Tribe has no tribal K-12 schools; parental involvement with public district is limited	CAB formed tribal–school partnership committee Principal and tribal liaisons are CAB members School initiatives shared at CAB meeting	CAB co-facilitated by school-based anti-bully and injury prevention director In-school dental exams and sealants Antismoking policies promoted

Abbreviations: CAB, community advisory board; UW, University of Wisconsin.

a Examples of primary CAB activities, not an exhaustive list of initiatives completed.

The CABs produced tangible results that require little cost to maintain, including a cultural playground, community and individual gardens, landscaped safe beach access, and gardening and canning workshops. In addition, policies related to food served at tribal meetings, Head Start, and school lunch rooms were changed to encourage more healthy options. Because of CAB interventions, the number of environmental supports in each community increased while environmental barriers decreased. CABs enabled community members and stakeholders to engage in regular intra-CAB networking, resulting in increased awareness and collaboration across agencies.

### CAB networking and dissemination

Tribes share similar histories, barriers to health (Box), and health risks. Thus inter-CAB networking was an important outcome of this work. If a barrier was identified (8) and successfully addressed by one CAB, the facilitator shared details of the issue, intervention design, and action with another CAB, if requested. Members from one CAB shared their expertise at meetings of other CABs.

National research results and grant opportunities were regularly shared at CAB meetings. For example, one of the CABs adopted findings from a national lunchroom design study detailing food and display placement affecting healthier selections. State and national funding opportunities introduced at CAB meetings were pursued, with several grants awarded and existing resources repurposed and leveraged to support CAB priorities. For example, one CAB was awarded a $50,000 grant from the Wisconsin Partnership Program to continue CAB work and develop a comprehensive data management and evaluation plan for youth obesity prevention. Members from each CAB represented the collaborative project at local and national forums to talk about process and results. Tribal and community newspapers regularly featured CAB work, and state media coverage increased awareness of the CABs (12).

### Academic engagement

Academic partners brought their expertise and university resources to the CABs. Once the CAB had identified environmental barriers, the academic partners helped develop plans for future research partnerships that were beneficial to both the university researchers and the tribe (13). For example, a University of Wisconsin (UW) faculty landscape architect and several UW students he recruited worked with tribal elders to design and install a culturally appropriate and environmentally friendly playground in Bad River with CAB members and volunteers from the community. Donations of natural resources (eg, acorns, shed antlers, sand, rocks, pinecones, logs, lumber) and equipment (eg, a canoe for harvesting wild rice, maple sugar pan, bird houses) were integrated into the project (Figure 2). CAB interventions also provided university students with internship opportunities in which they applied their knowledge and skills to work with the community.

A university researcher introduced his study at 2 CABs. The study logo was designed by a tribal member, and CAB members from the diabetes prevention program accompanied the researcher to tribal health committee meetings to gain approval. The CAB edited and simplified medical language for study recruitment and consent forms — an important CBPR milestone and an example of a CAB-enabled strategy that may increase trust and participation in future research.

## Interpretation

HCSF is one of the few family-based and community-based healthy lifestyle interventions in AI communities. Community and academic partners created successful CABs and CAB-led interventions in 3 rural Wisconsin AI reservations. At all 3 CABs, community engagement efforts and interventions decreased the number of environmental barriers to health and showed visible, practical results. Monthly CAB meetings became a key place for conversation in the community and empowered members to identify their own unique environmental barriers to health. A sense of group allegiance and responsibility was fostered. Together with tribal leadership, CABs became a driving force for change. And providing an important marker of success, CAB work continues to this date.

Insights into what made the CABs successful include providing a meal and a place to network in a professionally organized and facilitated meeting environment that set a tone for respect, equal communication, and mutual trust (11). Maintaining open meetings with ongoing recruitment and no predetermined size led to a continual infusion of fresh perspectives and energy. CAB success was also supported by trusting relationships between the facilitator and CAB members; presentations by local and academic experts, some of whom joined CABs as regular members; participation of experts who spent extended periods of time in communities to complete interventions; and use of student talent.

Academic support undeniably played a role in CAB success, including facilitation and coordination of meetings, recruitment, and financial support for meals and meeting space. Studies, news, and funding opportunities shared by academic partners were also critical in informing and leveraging CAB work. Offering academic assistance for ongoing tribal programs and grant writing showed good faith for ongoing community–academic partnership activities within and outside the CAB. In addition, academic and community presentations at national meetings helped increase CAB visibility, foster CAB success, and achieve sustainability. However, similar to findings by Cargo et al (14), the CABs were primarily driven by community needs, interests, and initiatives, with high levels of ongoing community engagement that were noted from the start.

The university and tribal partners initially identified concerns about attendance, focus, and sustaining commitment until tangible results were attained. The facilitator and CAB members observed that attendance was influenced positively by interest in agenda topics, guest speakers, meeting location, and tribal events and negatively by seasonal activities, weather, meeting location, other community events, politics, and other tribal meetings. Some CAB members shared that without academic leadership, they would have been unable to justify absence from their jobs. Some smaller tribal agencies could not send a representative to CAB meetings each month. However, they presented at specific meetings or served on informational panels.

After 4 years, when new member recruitment was transitioned from the facilitator to CAB members, the number of new attendees initially decreased, probably because of the absence of a specific person who focused on attendance. Conducting meetings in different tribal communities, as opposed to main tribal villages, would have resulted in different tribal representation at meetings. However, at this time, new CAB members continue to bring fresh perspectives and new environmental issues to discuss; many of the new members represent agencies not previously involved.

At times progress was slow because CABs raised historical tribal issues that communities had long struggled with to no avail. CABs took specific steps to sustain focus and momentum. For example, the CAB mission was printed on every meeting agenda and the facilitator began each meeting with a brief CAB overview. Introductory materials, including a list of accomplishments and current initiatives, were always available to attendees. The facilitator also met regularly with tribal leaders and with new and old partners to update them on CAB activities.

One of the most difficult issues in any community-based project is sustainability after the original intervention funding is exhausted. With regard to our project, all 3 communities have continued their CABs, with the Menominee CAB work absorbed by a larger community engagement committee. Although different in scope, each CAB continues as a self-sustaining, action-oriented team making substantial progress toward community change. Although academics continue to provide technical assistance and intervention support, academic funding and facilitation has not been necessary to sustainability. CAB members continue to be the primary drivers of community change.

The combined power of a community’s in-kind contributions and academic engagement in bringing people together for regular meetings should not be underestimated. It can mobilize and build tribal capacity to address chronic and evolving health issues and implement successful, sustainable environmental interventions. CABs can be highly effective action teams to improve community environments by expanding local food and activity choices and implementing policy changes that improve community health.

## References

[1] Barnes PM , Adams PF , Powell-Griner E . Health characteristics of the American Indian or Alaska Native adult population: United States, 2004–2008. Natl Health Stat Report 2010;(20):1–22. 20583451

[2] Centers for Disease Control and Prevention. Disparities in premature deaths from heart disease — 50 states and the District of Columbia, 2001. MMWR Morb Mortal Wkly Rep 2004;53(6):121–5. 14981360

[3] Cobb N , Wingo PA , Edwards BK . Introduction to the supplement on cancer in the American Indian and Alaska Native Populations in the United States. Cancer 2008;113(5 Suppl):1113–6. 10.1002/cncr.23729 18720369

[4] Indian Health Services. Regional differences in Indian health, 2002–2003. Washington (DC): US Department of Health and Human Services; 2009.

[5] Adams AK , Harvey H , Brown D . Constructs of health and environment inform child obesity prevention in American Indian communities. Obesity (Silver Spring) 2008;16(2):311–7. 10.1038/oby.2007.71 18239638

[6] LaRowe TL , Wubben DP , Cronin KA , Vannatter SM , Adams AK . Development of a culturally appropriate, home-based nutrition and physical activity curriculum for Wisconsin American Indian families. Prev Chronic Dis 2007;4(4):A109. http://www.cdc.gov/pcd/issues/2007/oct/07_0018.htm. Accessed January 15, 2014. 17875253 PMC2099274

[7] Adams AK , LaRowe TL , Cronin KA , Prince RJ , Wubben DP , Parker T , The Healthy Children, Strong Families intervention: design and community participation. J Prim Prev 2012;33(4):175–85. 10.1007/s10935-012-0275-y 22956296 PMC3904366

[8] Adams A . Understanding community and family barriers and supports to physical activity in American Indian children. J Public Health Manag Pract 2010;16(5):401–3. 10.1097/PHH.0b013e3181f51636 20689388 PMC3477811

[9] American Nutrition Association. USDA defines food deserts. Nutrition Digest 2011;36(3). http://americannutritionassociation.org/newsletter/usda-defines-food-deserts. Accessed January 15, 2014.

[10] Israel BA , Lichtenstein R , Lantz P , McGranaghan R , Allen A , Guzman JR . The Detroit Community–Academic Urban Research Center: development, implementation, and evaluation. J Public Health Manag Pract 2001;7(5):1–19. 10.1097/00124784-200107050-00003 11680026

[11] Wallerstein N , Duran B , Minkler M , Foley K . Developing and maintaining partnerships with communities. In: Israel BA, Eng E, Schulz AJ, Parker EA, editors. Methods in community-based participatory research for health. San Francisco (CA): Jossey-Bass; 2005; p. 31–51.

[12] Fisher M. Taking it outside. Grow — Wisconsin’s Magazine for the Life Sciences 2011;Spring:28–33.

[13] Adams A , Miller-Korth N , Brown D . Learning to work together: developing academic and community research partnerships. WMJ 2004;103(2):15–9. 15139553

[14] Cargo MD , Delormier T , Lévesque L , McComber AM , Macaulay AC . Community capacity as an “inside job”: evolution of perceived ownership within a university–Aboriginal community partnership. Am J Health Promot 2011;26(2):96–100. 10.4278/ajhp.091229-ARB-403 22040390

